# Use of Chinese Herb Medicine in Cancer Patients: A Survey in Southwestern China

**DOI:** 10.1155/2012/769042

**Published:** 2012-09-11

**Authors:** Tai-Guo Liu, Shao-Quan Xiong, Yan Yan, Hong Zhu, Cheng Yi

**Affiliations:** ^1^Department of Abdominal Cancer, West China Hospital, Sichuan University, Sichuan Province, Chengdu 610041, China; ^2^Department of Medical Oncology, Teaching Hospital of Chengdu University of Traditional Chinese Medicine, Sichuan Province, Chengdu 610075, China

## Abstract

Chinese herb medicine (CHM) is the most commonly reported traditional Chinese medicine (TCM) modality. This study aimed to assess the prevalence and associated factors of CHM use in cancer patients in southwestern China. Cancer patients from eleven comprehensive cancer centers were asked to complete a structured questionnaire. Of 587 available replies, 53.0% used CHM. Multiple logistic regression analysis showed that educational level, stage of disease, duration of cancer since diagnosis, marital status, and previous use of CHM were strongly associated with CHM use after cancer diagnosis. The source of information about CHM was mainly from media and friends/family. CHM products were used without any consultation with a TCM practitioner by 67.5% of users. The majority used CHM to improve their physical and emotional well-beings and to reduce cancer therapy-induced toxicities. About 4.5% patients reported side effects of CHM. This survey revealed a high prevalence of CHM use among cancer patients. However, these patients did not get sufficient consultation about the indications and contradictions of these drugs. It is imperative for oncologists to communicate with their cancer patients about the usage of CHM so as to avoid the potential side effects.

## 1. Introduction

Complementary alternative medicine (CAM) is becoming more and more popular all over the world. The use of CAM has increased steadily over the past two decades, and undoubtedly it has gained medical, economic, and sociological importance [1]. Traditional Chinese medicine (TCM) is an available option in many cancer centers in Asia [2], Western countries [3], and Africa [4]. TCM has been practiced in China for more than 2,000 years, and Chinese herb medicine (CHM) is the most commonly used category of TCM [5, 6]. It is based on the Chinese philosophy of Yin-Yang and Five Elements [7, 8]. It emphasizes the holistic principles and emphasizes harmony with the universe. The basic theories of TCM include five-zang organs and six-fu organs, qi (vital energy), blood, and meridians [9–11]. The introduction of Western medicine in the 17th century brought about significant changes in the development of TCM. Western medicine started to dominate the market. Currently, Western medicine and TCM are the two mainstream medical practices in China. Generally speaking, medical doctors in urban areas are more likely to use Western medicine, while TCM is practiced mainly in rural areas. 

Cancer is a major disease in China with great social and economic burden [12]. It is the leading death cause in urban China and the second one in rural China [13]. TCM and Western medicine are different in their etiological concepts and therapeutic approaches about cancer. According to Chinese medicine theory, cancer is the manifestation of a qi disturbance which may be treated by mobilizing qi. In Western medicine, cancer is defined as uncontrolled growth of malignant cells which may be treated with surgery, chemotherapy and radiotherapy. Although there is still much debate about the efficacy of CHM, more and more data have demonstrated that CHM has the potential to improve tumor response to chemotherapy as well as patient's survival rates [14–16]. Chinese herbs have an anticancer effect by inducing apoptosis of cancer cells, enhancing the immune system, inducing cell differentiation, and inhibiting telomerase activities and growth of tumors [17, 18]. In addition, a growing number of researches have indicated that Chinese herbs might reduce the toxicities of adjuvant therapies [19, 20]. In light of the aging population and ever increasing incidence of cancer, it is of great importance to investigate the CHM use in cancer patients.

Information on contemporary CHM use, attitudes, and beliefs is valuable for clinicians, decision makers, and patient educators who should respond to the growing interests among patients, particularly in comprehensive cancer centers. However, there have been no data of this sort in southwestern China. Therefore, we performed a survey about CHM use in China. Cancer patients in southwestern China generally represent the cancer patients in the underdeveloped area of China. Chinese people who live in the rest of the world always regard CHM as a choice of CAM therapies for many medical conditions. Research on the use of CAM, particularly the use of CHM among Chinese cancer patients, will not only provide insight in the current CHM use in cancer patients in China, the respective data could also be a source of information for future empirical and clinical studies. Therefore, we conducted this survey about the prevalence, influencing factors, reasons, source of information, and side-effects of CHM use in southwestern China.

## 2. Subjects and Methods

### 2.1. Participants and Settings

A descriptive survey design was used to collect data through a questionnaire about CHM therapies. All directors of comprehensive cancer centers in southwestern China were approached for possible collaboration. Eleven agreed to participate in the survey while 2 refused. Data were collected in the outpatient clinics of the 11 comprehensive cancer centers from June 2010 to August 2010. Both metastatic and nonmetastatic cancer patients who were at least 18 years of age were approached for possible inclusion in the study. Based on his/her interest and/or experience in CAM, a responsible physician was selected from each of these collaborating centers, who was called an investigator. Each investigator was responsible for introducing the study to all potential participants and then determining their eligibility for inclusion. As part of the consent process, patients were informed that they could withdraw from the study at any time and skip any survey question. To increase accuracy, patients recorded their responses directly onto the questionnaire. Questionnaires were returned to an investigator and coded with a unique identification number to ensure confidentiality. The investigator attempted to contact every patient in the clinic. They maintained a daily record of the accrual process, including the number of patients who could not be screened for eligibility because of the busy clinic environment and of those screened, and the reasons for ineligibility and nonparticipation. 

### 2.2. Questionnaire

In the present study, we used a modified version of questionnaire developed by Swisher et al. [21]. The topics in this questionnaire covered the prevalence, influencing factors, reasons, source of information, and side effects about CHM use. After a draft questionnaire was prepared, the questionnaire was reviewed by both Chinese medicine experts (*N* = 5) and Western medicine practitioners (*N* = 5), and then a pilot test was performed in a small group of patients in the outpatient clinic of the Department of Abdominal Cancer, West China Hospital, Sichuan University, after which the questionnaire was finalized. The original questionnaire we used was written in Chinese. The questionnaire attached of the supplementary material available online at doi: 10.1155/2012/769042 has been translated into English. The final questionnaire consisted of two parts, that is, the first part on the demographic information of participants (age, gender, educational level, household income, marital status and religions); and the second part on participants' clinical condition and use of CHM (activity of daily life, duration of cancer since diagnosis and stage of the cancer, methods of obtaining information about CHM, previous history of CHM use before cancer diagnosis, reasons for using CHM, and whether they used CHM after consulting a Chinese medical practitioner, as well as CHM's adverse effects). CHM users were defined as patients who had used CHM at least once since they were diagnosed with cancer, and those who never used CHM after cancer diagnosis were considered as nonusers. And CHM included raw herbal medicine (zhong yao cai), sliced herbal medicine (zhong yao yin pian), and patent medicine (zhong cheng yao).

### 2.3. Data Analysis

Differences in demographic and clinical characteristics between CHM users and nonusers were assessed using *χ* ^2^ test. Factors associated with CHM use were identified via multiple logistic regression analysis (*P* < 0.05). The analysis provided an odds ratio and a 95% CI for each variable while simultaneously controlling for the effects of other variables. Data were analyzed with SPSS 13.0 software. 

## 3. Results

### 3.1. Demographic and Clinical Characteristics of Study Participants

A total of 1,835 patients attended the clinics of the 11 cancer centers during the study period; however, 591 patients left the clinic before being invited to participate or screened by the investigators due to the busy clinic environment. Therefore, a total of 1,244 patients were screened for eligibility by the investigators, and 902 (72.5%) were eligible for participation. Of the 342 patients who were ineligible, reasons for exclusion included the following: without a cancer diagnosis (*n* = 166), younger than 18 years of age (*n* = 58), or unable to participate because of medical problems (*n* = 118).

Of the 902 eligible patients, 638 patients consented to participate of whom 51 were later excluded because they did not complete (*n* = 22) or return (*n* = 29) the questionnaire. Finally, a total of 587 patients consisting of 355 (60.5%) male and 232 (39.5%) female patients completed the survey with a response rate of 92.0% (587/638). Their mean age was 55.68 years; most participants (*n* = 533; 90.8%) were married, most earned <24,000 RMB annually (*n* = 378; 64.4%). Details of the demographic and clinical characteristics of all study participants are summarized in Table 1. 

### 3.2. CHM Use

Current CHM use was reported by 53.0% (*n* = 311) of the responders. Comparisons by *χ* ^2^ test showed that CHM users and nonusers did not differ with respect to age, gender, marital status, religious affiliation, and activity of daily living. CHM users differed from nonusers in terms of educational level, household income, stage of disease, duration of cancer since diagnosis, and previous history of CHM use. Details on CHM use of all study participants are summarizes in Table 1. The multiple logistic models indicated that patients with advanced stage of disease, or a previous history of CHM use, were more likely to use CHM after cancer diagnosis (Table 2). Patients with a higher educational level, a disease duration between 6 to 60 months, or got married were less likely to use CHM. 

### 3.3. Sources of Information about CHM

Approximately 50.5% of patients used CHM based on the recommendations from media (e.g., TV programs, newspapers, internet, radios), followed by family members or friends (48.4%). Only 32.5% of patients obtained information about CHM from the TCM practitioners. Other sources of information (8.6%) included personal knowledge and other patients who used CHM. About 74.0% of patients stated that they needed more information about CHM therapies from books and physicians.

### 3.4. Reasons for Using CHM

The most commonly reported reason for using CHM among the CHM users was a desire to improve physical and emotional well-beings and to reduce cancer therapy-induced toxicities (65.7%). They also used CHM because they wanted more control in the decisions about their medical care (46.2%) and believed that it was nontoxic (38.9%).

### 3.5. Side Effects

Fourteen patients (4.5%) reported side-effects of the CHM therapies they had used. These side-effects included gastric upset and nausea, stomachache and diarrhea. Three experienced serious side-effects, including renal failure and cardiac arrhythmias, possibly because they used CHM for a long time without consulting their doctors. Of them, one patient used Long Dan Xie Gan Decoction (zhong cheng yao) for a long time, which might be associated with his renal failure; two used large doses of Chansu (prepared from the skin and venom glands of the toad) which might be related with their cardiac arrhythmias. 

### 3.6. Reasons for Not Using CHM

Patients who did not use CHM were asked to indicate the reason. The majority (*n* = 229; 83.0%) reported that they were already satisfied with the efficacy of the conventional treatment. Other reasons included lack of information about CHM, inconvenience of CHM use, no such recommendations from their physicians, and inability to pay for CHM.

## 4. Discussion

Through the joint efforts of several generations of practitioners in TCM and integrated medicine of oncology, we have made some achievements in TCM cancer treatment, in terms of treatment concepts, methods, and laboratory and clinical research [22, 23]. Indeed, previous studies have shown that some CHMs like *Scutellaria baicalensis* and honokiol, as well as decoctions like Dang-Gui-Bu-Xai-Tang and anticancer number one, have potential anticancer effects [24–27]. However, very few studies have been conducted to investigate the CHM use in cancer patients in China. Therefore, we conducted such a survey in southwestern China.

Based on the limited data available, the prevalence of CHM use (53.0%) during cancer treatment in southwestern China seems to be comparable to that in other Chinese regions [28, 29], but much higher than that in other countries. For example, a survey in Turkey involving 615 adult cancer patients showed that 291 patients (47.3%) had ever used CAM which consisted mainly of herbal agents (95%) [30]. A survey in Norway including 120 cancer patients revealed that 37% to 38% of patients had used herbs during chemotherapy [31]. However, a survey in England including 1134 cancer patients indicated that only 19.7% of patients had ever used herbs [32].Natural health products were used by 26.5% of prostate cancer patients in a survey conducted in Canada[33], which is also much lower when compared with China.The different prevalence of herb use between China and other countiesmay bemainly due toa different socioculture andmedicalsystem in different ethnic groups.A study analyzed the types and the prevalence of CAM used by American women with breast cancer in four ethnic groups and found that Chinese-American women with breast cancerfavored herbal therapies much more and were less likely to use megavitamins than whites or blacks. The widespread use of CHM in the Chinese population might not be surprising since TCM has been practiced in China for more than 2,000 years, which has led to the so-called “TCM culture”. Also, use of CHM therapies may be related to the availability of such therapies in a given geographical setting [34].

Multivariate analysis revealed a close association between CHM use and educational level, stage of disease,duration of cancer since diagnosis, marital status, andprevious history of CHM use. In the present study, patients with a higher educational level were less likely to use CHM.But previous studies reported that a high educational level was a potential predictor of herb medicine use [35–37], which is not consistent with our findings.Due to the success of economic reform in the past 30 years, the concept of westernization is popular among Chinese people with higher education levels.However, Chinese philosophy of life is deeply engrained and rooted within the Chinese population. Many Chinese patients' perception of health, illnesses, and their treatment options were influenced by traditional Chinese cultural health beliefs, especially the elderly and those in rural areas, whose educational level is often lower.

Previous studiesfound that patientswith advanced stage of diseasewere more prone to use CHM [34, 37]. Similar results were obtained in our study. This is possibly because theyhave little hope from conventional treatments and often experience serious adverse effects of conventional therapies, then turning to CHM as an additional intervention to improve the quality of their lives. The majority of patients (65.7%) in ourstudy believed that CHM could improve their well-being and ameliorate side effect caused by cancer itself or its treatment. 

Cancer patients obtained the information about CHM through a wide variety of sources before they began CHM therapy. Media,including internet, TV, newspaper, and radios, is the most important source of information. This may be problematic, as some media may exaggerate treatment effect, even provide misleading information about herb medicines [38]. Friends and family members are also important information sources. Since they may have experienced the efficacy of CHM, they recommend CHM to cancer patients in his/her family. It is interesting to note that the role of physicians as a source of information is pretty much ignored, which is consistentwith the findings from previous studies [36, 37]. These results perhaps reflect the disapproval of CHM therapies by the medical community or the lack of information to the medical community about the available and effective CHM therapies. Patients should obtain information about CHM from more reputable organizations, and be encouraged to receive CHM therapies whose effectiveness and safety have been well validated. 

Based on thousands of years of clinical experience, CHM is generally safe when taken under the guidance of a skilled physician. Adverse effects and toxicities usually occur due to a lack of knowledge about CHM. In our study, some patients suffered side effects because they used CHM by themselves without sufficient information about these medicines. Since most of sliced herbal medicines and patent medicines are classified as over-the-counter (OTC) drugs in China. Many cancer patients self-take them without consulting a qualified Chinese medical practitioner.

CAM use in cancer patients is increasing throughout the world and herb medicine comprises an important part of CAM. CHM has been used for thousands of years in China and abundant experiences in the treatment of cancer have accumulated. Besides,many cancer patients in other countries (e.g., American, England, Norway, Australia, Canadian, and Thailand) also began to use herb medicine [30–32, 37, 39, 40]. Since most of the cancer patients who used herb medicine for their cancer treatment did not get enough education about the indications and contradictions of these herbs, the therapeutic effects of these drugs may be reduced and many side-effects may occur. 

## 5. Conclusion

This survey revealed a high prevalence of CHM use among cancer patients insouthwestern China. However, most patients did not receive sufficient consultation about the indication and side-effects of these drugs. Thus, it is imperative that oncologists educate their cancer patients about the potential benefits and side effects of CHM therapies, remind them of the potential risks of self-taking CHM, and suggest them to use CHM under the supervision of qualified Chinese medical practitioners.

## Supplementary Material

The Supplementary Material is the questionnaire we used in the survey. The topics in this questionnaire covered the prevalence, in*ﬂ*uencing factors, reasons, source of information, and side effects about CHM use.

## Figures and Tables

**Table 1:** Demographic characteristics and CHM use.

Background	No. of patients	No. of Users	(%)	No. of nonusers	(%)	*P* (*χ* ^2^ test)
Total	587	311	(52.98)	276	(47.02)	
Age (years)						0.53
<40	111	64	(57.66)	47	(42.34)	
40–60	290	149	(51.38)	141	(48.62)	
>60	186	98	(52.69)	88	(47.31)	
Gender						0.30
Male	355	182	(51.27)	173	(48.73)	
Female	232	129	(55.60)	103	(44.40)	
Marital status						0.51
Married	533	286	(53.66)	247	(46.34)	
Never	28	12	(42.86)	16	(57.14)	
Others	26	13	(50.00)	13	(50.00)	
Activity of daily living						0.10
Free	181	85	(46.96)	96	(53.04)	
Somewhat limited	249	143	(57.43)	106	(42.57)	
Bed rest (≥50% of each day)	157	83	(52.87)	74	(47.13)	
Education (years)						0.02
0	23	12	(52.17)	11	(47.83)	
−9	201	99	(49.25)	102	(50.75)	
−12	179	85	(47.49)	94	(52.51)	
≥13	184	115	(62.50)	69	(37.50)	
Practicing religion						0.12
No	496	256	(51.61)	240	(48.39)	
Yes	91	55	(60.44)	36	(39.56)	
Annual income (RMB)						0.00
−24000	378	183	(48.41)	195	(51.59)	
−60000	167	104	(62.28)	63	(37.72)	
−120000	32	21	(65.63)	11	(34.38)	
>120000	10	3	(30.00)	7	(70.00)	
Previous history of CHM use						0.00
No	298	83	(27.85)	215	(72.15)	
Yes	289	228	(78.89)	61	(21.11)	
Staging						0.00
Early stage	305	176	(57.70)	129	(42.30)	
Advanced stage	282	135	(47.87)	147	(52.13)	
Duration of cancer since diagnosis (months)						0.00
−6	339	140	(41.30)	199	(58.70)	
−24	161	99	(61.49)	62	(38.51)	
−60	57	48	(84.21)	9	(15.79)	
>60	30	24	(80.00)	6	(20.00)	

**Table 2:** Factors associated with CHM use in the multivariate logistic regression.

Background	Odds ratio	95% CI
Age (years)		
<40	1.00	Reference
40–60	1.44	0.74−2.81
>60	0.88	0.54−1.44
Gender		
Male	1.00	Reference
Female	1.12	0.72−1.75
Marital status		
Never	1.00	Reference
Married	0.70	0.50−0.98
Others	1.16	0.43−3.14
Activity of daily living		
Free	1.00	Reference
Somewhat limited	0.83	0.23−2.97
Bed rest (≥50% of each day)	1.03	0.17−6.08
Education (years)		
0	1.00	Reference
−9	0.23	0.06−0.80
−12	0.54	0.31−0.94
≥13	0.46	0.27−0.79
Practicing religion		
No	1.00	Reference
Yes	1.49	0.81−2.74
Annual income (RMB)		
−24000	1.00	Reference
−60000	3.01	0.47−19.13
−120000	3.42	0.53−22.11
>120000	3.50	0.46−26.46
Previous history of CHM use		
No	1.00	Reference
Yes	10.69	6.94−16.45
Staging		
Early stage	1.00	Reference
Advanced stage	2.56	1.35−3.90
Duration of cancer since diagnosis (months)		
−6	1.00	Reference
−24	0.13	0.04−0.38
−60	0.32	0.10−0.99
>60	0.92	0.24−3.48

## References

[1] Chan A, Lin TH, Shih V, Ching TH, Chiang J (2012). Clinical outcomes for cancer patients using complementary and alternative medicine. *Alternative Therapies in Health and Medicine*.

[2] Hyodo I, Amano N, Eguchi K (2005). Nationwide survey on complementary and alternative medicine in cancer patients in Japan. *Journal of Clinical Oncology*.

[3] Swarup AB, Barrett W, Jazieh AR (2006). The use of complementary and alternative medicine by cancer patients undergoing radiation therapy. *American Journal of Clinical Oncology*.

[4] Ezeome ER, Anarado AN (2007). Use of complementary and alternative medicine by cancer patients at the University of Nigeria Teaching Hospital, Enugu, Nigeria. *BMC Complementary and Alternative Medicine*.

[5] Chen Z, Gu K, Zheng Y, Zheng W, Lu W, Shu XO (2008). The use of complementary and alternative medicine among Chinese women with breast cancer. *Journal of Alternative and Complementary Medicine*.

[6] Teng L, Jin K, He K (2010). Use of complementary and alternative medicine by cancer patients at zhejiang university teaching hospital zhuji hospital, China. *African Journal of Traditional, Complementary and Alternative Medicines*.

[7] Patwardhan B, Warude D, Pushpangadan P, Bhatt N (2005). Ayurveda and traditional Chinese medicine: a comparative overview. *Evidence-Based Complementary and Alternative Medicine*.

[8] Lukman S, He Y, Hui SC (2007). Computational methods for Traditional Chinese Medicine: a survey. *Computer Methods and Programs in Biomedicine*.

[9] Seki K, Chisaka M, Eriguchi M (2005). An attempt to integrate Western and Chinese medicine: rationale for applying Chinese medicine as chronotherapy against cancer. *Biomedicine and Pharmacotherapy*.

[10] Zhou X, Peng Y, Liu B (2010). Text mining for traditional Chinese medical knowledge discovery: a survey. *Journal of Biomedical Informatics*.

[11] Liu J, Chen Z (2011). Traditional Chinese medicine in the new century. *Frontiers of Medicine*.

[12] Yang L, Parkin DM, Ferlay J, Li L, Chen Y (2005). Estimates of cancer incidence in China for 2000 and projections for 2005. *Cancer Epidemiology Biomarkers and Prevention*.

[13] Zhao P, Dai M, Chen W, Li N (2010). Cancer trends in China. *Japanese Journal of Clinical Oncology*.

[14] Shu X, McCulloch M, Xiao H, Broffman M, Gao J (2005). Chinese herbal medicine and chemotherapy in the treatment of hepatocellular carcinoma: a meta-analysis of randomized controlled trials. *Integrative Cancer Therapies*.

[15] Cho WCS, Chen HY (2009). Clinical efficacy of traditional chinese medicine as a concomitant therapy for nasopharyngeal carcinoma: a systematic review and meta-analysis. *Cancer Investigation*.

[16] McCulloch M, See C, Shu XJ (2006). Astragalus-based Chinese herbs and platinum-based chemotherapy for advanced non-small-cell lung cancer: meta-analysis of randomized trials. *Journal of Clinical Oncology*.

[17] Ruan WJ, Lai MD, Zhou JG (2006). Anticancer effects of Chinese herbal medicine, science or myth?. *Journal of Zhejiang University. Science. B*.

[18] Ruan M, Ji T, Yang W (2008). Growth inhibition and induction of apoptosis in human oral squamous cell carcinoma Tca-8113 cell lines by Shikonin was partly through the inactivation of NF-*κ*B pathway. *Phytotherapy Research*.

[19] Mori K, Kondo T, Kamiyama Y, Kano Y, Tominaga K (2003). Preventive effect of Kampo medicine (Hangeshashin-to) against irinotecan-induced diarrhea in advanced non-small-cell lung cancer. *Cancer Chemotherapy and Pharmacology*.

[20] Cho CS (2010). *Supportive Cancer Care with Chinese Medicine*.

[21] Swisher EM, Cohn DE, Goff BA (2002). Use of complementary and alternative medicine among women with gynecologic cancers. *Gynecologic Oncology*.

[22] Lin H, Liu J, Zhang Y (2011). Developments in cancer prevention and treatment using traditional Chinese medicine. *Frontiers of Medicine*.

[23] Guo Z, Jia X, Liu JP, Liao J, Yang Y (2012). Herbal medicines for advanced colorectal cancer. *Cochrane Database of Systematic Reviews*.

[24] Gao J, Morgan WA, Sanchez-Medina A, Corcoran O (2011). The ethanol extract of Scutellaria baicalensis and the active compounds induce cell cycle arrest and apoptosis including upregulation of p53 and Bax in human lung cancer cells. *Toxicology and Applied Pharmacology*.

[25] Ahn KS, Sethi G, Shishodia S, Sung B, Arbiser JL, Aggarwal BB (2006). Honokiol potentiates apoptosis, suppresses osteoclastogenesis, and inhibits invasion through modulation of nuclear factor-*κ*B activation pathway. *Molecular Cancer Research*.

[26] Hsieh CC, Lin WC, Lee MR (2003). Dang-Gui-Bu-Xai-Tang modulated the immunity of tumor bearing mice. *Immunopharmacology and Immunotoxicology*.

[27] Hong-Fen L, Waisman T, Maimon Y, Shakhar K, Rosenne E, Ben-Eliyahu S (2001). The effects of a Chinese herb formula, anti-cancer number one (ACNO), on NK cell activity and tumor metastasis in rats. *International Immunopharmacology*.

[28] Yang C, Chien LY, Tai CJ (2008). Use of complementary and alternative medicine among patients with cancer receiving outpatient chemotherapy in Taiwan. *Journal of Alternative and Complementary Medicine*.

[29] Lam YC, Cheng CW, Peng H, Law CK, Huang X, Bian Z (2009). Cancer patients’ attitudes towards Chinese medicine: a Hong Kong survey. *Chinese Medicine*.

[30] Tas F, Ustuner Z, Can G (2005). The prevalence and determinants of the use of complementary and alternative medicine in adult Turkish cancer patients. *Acta Oncologica*.

[31] Engdal S, Steinsbekk A, Klepp O, Nilsen OG (2008). Herbal use among cancer patients during palliative or curative chemotherapy treatment in Norway. *Supportive Care in Cancer*.

[32] Damery S, Gratus C, Grieve R (2011). The use of herbal medicines by people with cancer: a cross-sectional survey. *British Journal of Cancer*.

[33] Boon H, Westlake K, Stewart M (2003). Use of complementary/alternative medicine by men diagnosed with prostate cancer: prevalence and characteristics. *Urology*.

[34] Molassiotis A, Fernandez-Ortega P, Pud D (2005). Use of complementary and alternative medicine in cancer patients: a European survey. *Annals of Oncology*.

[35] Ferro MA, Leis A, Doll R, Chiu L, Chung M, Barroetavena MC (2007). The impact of acculturation on the use of traditional Chinese medicine in newly diagnosed Chinese cancer patients. *Supportive Care in Cancer*.

[36] Richardson MA, Sanders T, Palmer JL, Greisinger A, Singletary SE (2000). Complementary/alternative medicine use in a comprehensive cancer center and the implications for oncology. *Journal of Clinical Oncology*.

[37] Puataweepong P, Sutheechet N, Ratanamongkol P (2012). A survey of complementary and alternative medicine use in cancer patients treated with radiotherapy in Thailand. *Evidence-Based Complementary and Alternative Medicine*.

[38] Molassiotis A, Xu M (2004). Quality and safety issues of web-based information about herbal medicines in the treatment of cancer. *Complementary Therapies in Medicine*.

[39] Rausch SM, Winegardner F, Kruk KM (2011). Complementary and alternative medicine: use and disclosure in radiation oncology community practice. *Supportive Care in Cancer*.

[40] Oh B, Butow P, Mullan B (2010). The use and perceived benefits resulting from the use of complementary and alternative medicine by cancer patients in Australia. *Asia-Pacific Journal of Clinical Oncology*.

