# Supplementary material for: Use of Chinese Herb Medicine in Cancer Patients: A Survey in Southwestern China
Source: Evid Based Complement Alternat Med. 2012 Sep 11;2012:769042. doi: 10.1155/2012/769042 (PMC3446813; doi:10.1155/2012/769042)
Supplement: Supplementary file 1 — The Supplementary Material is the questionnaire we used in the survey. The topics in this questionnaire covered the prevalence, inﬂuencing factors, reasons, source of information, and side effects about CHM use. [file 769042.f1.doc]

Patient ID:

1. When was your cancer diagnosed?
 Year month

2. How old are you?
 Year month

3. Please indicate your sex.
 Male/Female

4. What is your current marital status?

(1)Never (2) Married (3) Others

5. Please indicate your education years.

(1)0 (2)1~9 (3)10~12 (4)>=13

6. Are you committed to any religion?

Yes/No

7. Your annual income (RMB) is?
 (1)<24000 (2)24000-60000

(3)60000-120000 (4)>120000

8. Is your present daily activity limited by the disease? Please tick the number below.
 (1)Free (2) Somewhat limited with slight symptoms

(3)Bed rest more than 50% of the day

9. Your stage of cancer?

(1)Early stage (2) Advanced stage

10. Have you ever used Chinese Herb Medicine (CHM)?(CHM includes raw herbal medicine, sliced herbal medicine and patient medicine)

Yes/No

11. Are you using CHM now?(CHM includes raw herbal medicine，sliced herbal medicine and patient medicine)

Yes/No

12. If “no”, why?

(1)Satisfied with the conventional treatment

(2)Lack of information about CHM

(3) Inconvenience use of CHM

(4)Physicians not recommend it

(5)Inability to pay for it

13. If “yes”, why did you start to use CHM?
 (1)Improve well-being and reduce toxicity (2) Control your disease

(3)Non-toxic

14. Did you experience any adverse effects from CHM?
 Yes/No

15. If “yes”, please specify:

16. What kinds of CHM led to these adverse effects?

17. Where did you get the information about CHM?
 (1)Media (such as the Internet, TV, newspaper, or radio)
 (2) Family members/Friends

(3)TCM practitioners

(4)Others such as personal knowledge and other patients

18. Do you want to get more information about CHM therapies from books and physicians?

Yes/No
